# Clinical Relevance of the Serial Measurement of Krebs von den Lungen-6 Levels in Patients with Systemic Sclerosis-Associated Interstitial Lung Disease

**DOI:** 10.3390/diagnostics11112007

**Published:** 2021-10-28

**Authors:** Yuichiro Shirai, Ryosuke Fukue, Yuko Kaneko, Masataka Kuwana

**Affiliations:** 1 Department of Allergy and Rheumatology, Nippon Medical School Graduate School of Medicine, Tokyo 113-8603, Japan; y-shirai@nms.ac.jp (Y.S.); r-fukue@nms.ac.jp (R.F.); 2 Scleroderma/Myositis Center of Excellence, Nippon Medical School Hospital, Tokyo 113-8603, Japan; 3 Division of Rheumatology, Department of Internal Medicine, Keio University School of Medicine, Tokyo 160-8582, Japan; ykaneko.z6@keio.jp

**Keywords:** biomarker, immunomodulatory treatment, interstitial lung disease, pneumoprotein, systemic sclerosis, scleroderma

## Abstract

Krebs von den Lungen-6 (KL-6) levels measured at baseline have been reported as a circulating biomarker useful for the detection, evaluation of severity and assessment of risk of the progression of interstitial lung disease (ILD) in patients with systemic sclerosis (SSc). In this retrospective study, longitudinal changes in serum KL-6 levels over 2 years were examined in 110 patients with SSc using prospectively collected cohort data. Serum KL-6 levels fluctuated in a significant proportion of the patients but remained stable in the remaining patients. A wide range of variability of longitudinal KL-6 levels was associated with the presence of ILD, diffuse cutaneous SSc, positive anti-topoisomerase I antibodies, negative anticentromere antibodies, increased ILD extent on high-resolution computed tomography, extensive disease, low pulmonary function parameters, high KL-6 levels at baseline and immunomodulatory treatment. Extensive disease was consistently identified as an independent factor associated with variability in KL-6 levels in different models of multiple regression analysis. We failed to demonstrate correlations between trends for KL-6 level changes during the 6 months after SSc diagnosis and ILD progression over 2 years in patients with SSc-ILD. Serum KL-6 levels fluctuate in SSc patients with ILD, especially in those with extensive disease, but the clinical utility of a serial KL-6 level measurement remains uncertain.

## 1. Introduction

Interstitial lung disease (ILD) is the leading cause of disease-related morbidity and mortality in patients with systemic sclerosis (SSc) [1]. While cyclophosphamide (CYC) and mycophenolate mofetil (MMF) are still mainstay treatments for SSc-ILD [2,3], nintedanib and tocilizumab have emerged as promising treatment options based on their efficacy in reducing the decline in pulmonary function shown in placebo-controlled, randomized controlled trials [4,5]. Ideally, therapeutic intervention for SSc-ILD should be introduced in patients at risk of progression at the timing of preserved pulmonary function [6]. However, in clinical practice, it is difficult to judge whether pharmacological treatment is necessary in individual cases because of the heterogeneous and unpredictable clinical course of SSc-ILD. Specifically, a substantial portion of patients show a progressive decline in pulmonary function, leading to end-stage lung disease (ESLD) and mortality, whereas others show stable pulmonary function during the entire course of the disease [7,8,9]. In this case, it is necessary to identify risk factors that predict a future progression of ILD early in the course of the disease. Demographic and clinical features, serum biomarkers, pulmonary function test (PFT) parameters and imaging characteristics have been reported as potential risk factors for progression in patients with SSc-ILD, but the majority of these features have not been verified in independent studies [10,11].

Krebs von den Lungen-6 (KL-6) is a sialylated chain of mucin 1, a high molecular weight glycoprotein expressed on the surface of type II alveolar epithelial cells [12]. KL-6 is known as a circulating pneumoprotein associated with lung parenchymal injury for various forms of ILD. An increase in circulating KL-6 levels is thought to result from the regeneration of type II alveolar epithelial cells and enhanced permeability due to the destruction of the air–blood barrier [12]. The utility of serum KL-6 levels in the diagnosis, evaluation of severity and assessment of risk of the progression of ILD have been reported in SSc patients (reviewed in [13,14]). Serum KL-6 levels are elevated in SSc patients with ILD compared with SSc patients without ILD and are inversely correlated with a forced vital capacity (FVC) and a diffusing capacity for carbon monoxide (DL_CO_) in patients with SSc-ILD. There are only a few studies assessing the roles of baseline KL-6 levels in predicting the progression of ILD. The elevated serum KL-6 levels at a diagnosis of SSc-ILD are associated with a subsequent decline in FVC and the future development of ESLD [8,15,16]. Thus, one can hypothesize that a short-term increase in serum KL-6 levels might reflect the increased activity of the pathogenic process of SSc-ILD and is potentially useful to predict subsequent ILD progression. However, little information on the longitudinal changes in serum KL-6 levels is available to date. Serum KL-6 levels were reported to decrease after treatment with immunosuppressants [16,17], but the roles of the change in KL-6 levels as a biomarker for disease activity and treatment response remain unclear. In this study, we retrospectively examined the variability of serial serum KL-6 levels using longitudinal serum KL-6 levels that were measured every 2 months in prospectively collected SSc cohorts. A potential role of short-term trends in KL-6 levels in predicting the subsequent progression of ILD was also investigated.

## 2. Materials and Methods

### 2.1. Study Population

In this retrospective study, 110 patients were selected from the SSc database at Keio University Hospital and Nippon Medical School Hospital since January 2007 based on (i) the fulfillment of the 2013 American College of Rheumatology/European League Against Rheumatism classification criteria for SSc [18], (ii) disease duration from the first non-Raynaud’s symptoms attributable to SSc ≤ 7 years at SSc diagnosis, (iii) the availability of chest X-ray and PFT data at SSc diagnosis, (iv) the availability of KL-6 level measurements obtained every 2 months for at least 6 months after SSc diagnosis and (v) the availability of follow-up data for ≥2 years unless mortality was related to ILD. Exclusion criterion included ESLD, which was defined as SSc-ILD resulting in at least one of the following: an FVC < 50%, a requirement for continuous oxygen supplementation or ILD-related death [8], at SSc diagnosis. For the analysis of potential correlations of short-term KL-6 level changes with the subsequent progression of ILD, 64 patients with SSc-ILD were selected from the original cohort based on the availability of follow-up PFT data at 2 years (24 ± 6 months).

### 2.2. Clinical and Laboratory Data

In the SSc database, a complete medical history, physical examination and laboratory evaluations were performed for each patient at the time of enrollment, and limited evaluations were conducted at each follow-up visit. The clinical data were prospectively and consecutively registered in the database. We collected the following data at SSc diagnosis for each patient: sex, age at study entry, disease duration from the first appearance of non-Raynaud’s symptoms attributable to SSc, diffuse cutaneous SSc (dcSSc) or limited cutaneous SSc and current or past smoker. FVC and DL_CO_ were measured using spirometry [19] and a single-breath method, respectively, and the final data were expressed as percentages of the predicted values. All patients took chest X-ray at SSc diagnosis and underwent chest high-resolution computed tomography (HRCT) when there were findings suggestive of ILD. The presence of ILD was defined based on radiological findings on HRCT [20] at SSc diagnosis. The extent of ILD was calculated using chest high-resolution computed tomography (HRCT), and ILD was classified as limited or extensive disease [21]. The immunomodulatory treatment for SSc-ILD was defined as the continuous administration of intravenous CYC for at least 6 courses [22], oral CYC and MMF for ≥12 months [23,24] and tocilizumab for ≥1 year [5]. SSc-related autoantibodies were identified using commercially available immunoassays and/or immunoprecipitation assays [25]. C-reactive protein was quantitatively measured using a latex coagulating nephelometry. Serum KL-6 levels were measured by an assay system using a latex-fixed anti-KL-6 monoclonal antibody with an automated analyzer (Nanopia KL-6; Sekisui Medical Co. Ltd., Tokyo, Japan).

### 2.3. Trends in Serum KL-6 Level Changes

Serum KL-6 levels were available for every 2-month interval. The coefficient of variation (CV) was used to assess the variability of longitudinal KL-6 levels over 2 years in individual patients. A short-term change in KL-6 levels was also examined for its potential utility to predict the future progression of ILD. For this analysis, trends in serum KL-6 level changes during the first 6 months after SSc diagnosis were used according to the European consensus statement on SSc-ILD that recommends careful monitoring every 3–6 months [2]. We used the following indices for the short-term KL-6 level changes: a regression coefficient that was calculated based on KL-6 levels at 4 time points (baseline and 2, 4 and 6 months); a ratio of the KL-6 levels at 6 months to the baseline; an area under the curve (AUC) above the standard line; an AUC above a line of the baseline KL-6 level; and the presence or absence of a consecutive rise during the 4 time point measurements.

### 2.4. ILD Progression

Clinically meaningful progression of ILD was defined as a ≥10% relative decline in FVC or a ≥5% to <10% relative decline in FVC and a ≥15% relative decline in DL_CO_ over a 2-year period, according to a report from the working group under the aegis of the Outcome Measures in Rheumatology (OMERACT) initiative [26]. In addition, a relative decline in FVC ≥ 10% over one year was used as an alternative outcome measure for progressive ILD [4,5,27]. The annual FVC change was calculated from data over a 2-year period. We also used the definition of progressive fibrosing ILD (PF-ILD) over 24 months, proposed by the consensus recommendations of an expert group of ILD physicians [28].

### 2.5. Statistical Analysis

Continuous variables are shown as the mean ± standard deviation (SD) and were compared between the groups using the Mann–Whitney U test. Categorical variables were compared using Fisher’s exact test or a chi-square test when appropriate. The correlation coefficient (r) was calculated using Spearman’s regression model. Simple regression analysis was used to identify clinical characteristics associated with the CV of serial serum KL-6 levels. Variables selected by simple regression analysis were further subjected to multiple regression analysis, in which variables that were highly relevant to each other were used in separate models. High relevance was defined as any correlation coefficient > 0.7 [29], a condition index in the diagnosis of multicollinearity > 15 [30] or variables that were known to be mutually exclusive. Finally, univariable logistic regression analysis was carried out to investigate whether KL-6 levels at baseline or short-term KL-6 level changes were associated with ILD progression over 2 years. Multivariable logistic regression analysis was further conducted using any immunomodulatory treatment as a confounding factor. All statistical analyses were performed using SPSS 25.0 statistical software (IBM; Chicago, IL, USA).

## 3. Results

### 3.1. Patient Characteristics at Baseline

Of the 362 patients with SSc registered in the SSc database of two hospitals, 110 patients were enrolled in this study (Supplemental Figure S1). The major reasons for exclusion included disease duration > 7 years at SSc diagnosis, the unavailability of a chest X-ray and a PFT at SSc diagnosis due to a referral to our hospitals after diagnosis and treatment conducted at other hospitals and a lack of regular hospital visits every 2 months. Twenty-one patients were excluded because of a follow-up period < 2 years. Of these, two patients died of colon cancer or acute lung injury related to an influenza virus infection within 2 years of SSc diagnosis. The loss of follow-up or an insufficient follow-up period was the reason for the remaining patients.

Table 1 shows the baseline characteristics and immunomodulatory treatment conducted during the 2 years after SSc diagnosis in the 110 patients with SSc. In the entire SSc population, the disease duration was 2.6 ± 2.0 years, and 42% of them had dcSSc. The most common SSc-related autoantibody was anti-topoisomerase I (topo I), which was detected in 41% of the patients. Seventy-six patients took chest HRCT, and 71 (93%) had ILD at baseline. Thirty-nine patients (37%) received immunomodulatory treatment for 2 years after their SSc diagnosis. For the analysis of potential correlations of serial KL-6 level changes with ILD progression, we selected 64 patients with SSc-ILD from the entire 110 SSc patients based on the presence of ILD by HRCT and the availability of follow-up PFT data at 2 years. These patients had trends toward higher frequencies of diffuse cutaneous SSc (dcSSc) and anti-topoisomerase I (topo I) antibodies and had lower FVC and DL_CO_ and higher baseline KL-6 levels than the entire SSc population.

### 3.2. Factors Associated with the Variability of Serial KL-6 Levels

The distribution of serum KL-6 levels at SSc diagnosis was highly variable, ranging from 149.8 to 4251.0 U/mL. The serum KL-6 levels changed over time in a group of patients but remained stable in the remaining patients; the CV over 2 years ranged from 0.055 to 0.473. We first examined the factors associated with the CV of serial KL-6 levels in the 110 patients with SSc (Table 2). As a result, the presence of ILD, dcSSc, positive anti-topo I antibodies, negative anticentromere antibodies, an increased ILD extent shown on HRCT, extensive disease, low FVC, low DL_CO_, a high KL-6 level at baseline and any immunomodulatory treatment were associated with a wide range of variability.

**Figure 1:**
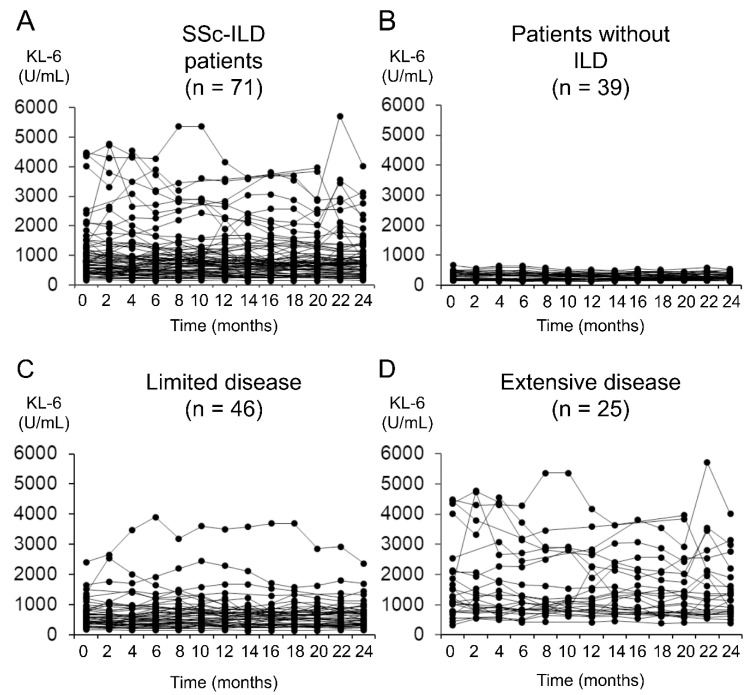
Longitudinal changes in serum KL-6 levels over 2 years after SSc diagnosis. (**A**) SSc patients with ILD; (**B**) SSc patients without ILD; (**C**) SSc patients with ILD and limited disease; and (**D**) SSc patients with ILD and extensive disease.

**Figure 2:**
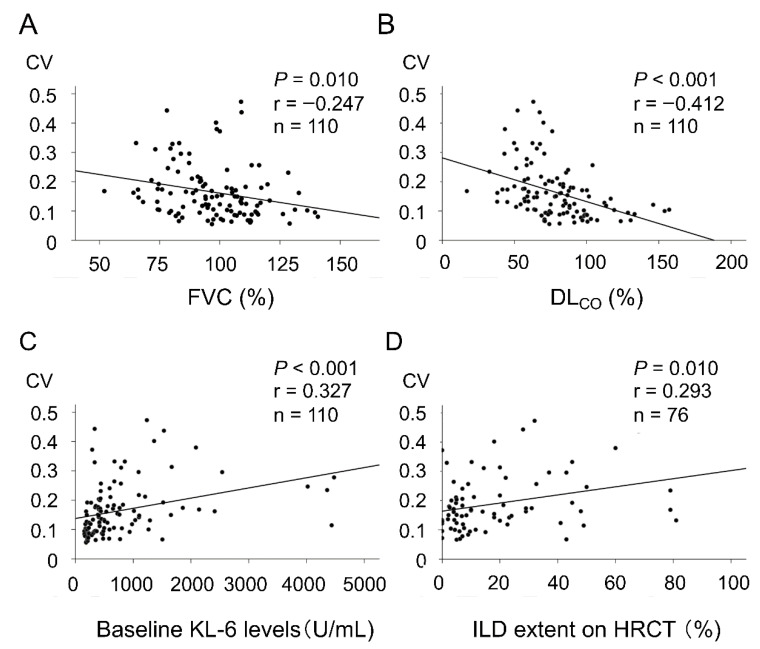
Correlations between the coefficient of variation (CV) of serial KL-6 level changes and (**A**) FVC, (**B**) DL_CO_ and (**C**) baseline KL-6 levels in 110 patients with SSc or (**D**) ILD extent on HRCT in 76 patients who underwent chest HRCT at SSc diagnosis.

When the SSc patients were divided into two groups based on the presence or absence of ILD, the serum KL-6 levels fluctuated over 2 years in the majority of the SSc patients with ILD (Figure 1A). In contrast, the patients without ILD showed consistently low KL-6 levels without fluctuations (Figure 1B). In the SSc patients with ILD, the fluctuations in the KL-6 levels were more prominent in the patients with extensive disease than in those with limited disease (Figure 1C,D). In addition, the CV of the serial KL-6 level changes was significantly negatively correlated with FVC and DL_CO_ and positively correlated with KL-6 levels at baseline (Figure 2A–C). In the 76 patients who underwent chest HRCT at SSc diagnosis, there was a significant correlation between the CV of the serial KL-6 level changes and the ILD extent shown on HRCT (Figure 2D).

The majority of factors associated with the wide range of variability in serial KL-6 levels were related to the presence or severity of ILD. Thus, multiple regression analysis was conducted using factors that were identified using the simple regression model, which were adjusted for sex, age and smoking history as cofounding factors (Supplemental Table S1). We created a total of 12 models by considering the combinations of high relevance variables, including the disease extent shown on HRCT and extensive disease; positive anti-topo I and negative anticentromere antibodies; the presence of ILD and FVC; and the presence of ILD and DL_CO_. Extensive disease was consistently identified as an independent factor that was associated with a wide range of fluctuations in the serial KL-6 levels.

### 3.3. Potential Utility of Short-Term KL-6 Change in Predicting ILD Progression

During the 2 years of follow-up in 64 patients with SSc-ILD, 30 (47%) received immunomodulatory treatment, including intravenous CYC (n = 11), oral CYC (n = 6), MMF (n = 6) and tocilizumab (n = 8). One patient received intravenous CYC followed by tocilizumab. The majority of the patients had a stable FVC during the 2 years of follow-up, while a few patients experienced a decline in FVC (Supplemental Figure S2). Clinically meaningful ILD progression, as defined by the OMERACT criteria, over 2 years was observed in 12 patients (19%), while only two (3%) experienced a relative decline in FVC ≥ 10% over one year. On the other hand, 22 patients (34%) developed PF-ILD over the 2 years of follow-up. In the following analysis, a relative decline in FVC ≥ 10% over one year was not used as the index for ILD progression due to the small number of patients who met this definition.

We compared the clinical characteristics between the patients who experienced a progression of ILD defined by the OMERACT criteria (Table 3). The patients who experienced ILD progression had a higher ILD extent shown on HRCT and a higher prevalence of extensive disease than the patients without ILD progression (*p* = 0.041 and 0.020, respectively). Extensive disease at baseline was again more common in the patients who developed PF-ILD than in those who did not (*p* = 0.031) (Supplemental Table S2).

Finally, we conducted univariate logistic regression analysis to examine whether the baseline KL-6 levels or the short-term changes in KL-6 levels during the first 6 months were useful to predict ILD progression in the subsequent 2 years. As a consequence, the baseline KL-6 levels or any of the trends for the short-term KL-6 level changes were not associated with a progression of ILD as defined by the OMERACT criteria or PF-ILD (Table 4). The additional analysis that was adjusted for any immunomodulatory treatment failed to show any significant correlation (Supplemental Table S3).

## 4. Discussion

We have demonstrated that serum KL-6 levels fluctuate during the course of SSc using longitudinal data of serum KL-6 levels measured every 2 months for 2 years. A high range of fluctuations in KL-6 levels was associated with ILD, especially ILD with a broader disease extent and impaired pulmonary function, suggesting that the serial measurement of serum KL-6 levels might detect changes in the ongoing pathogenic process of SSc-ILD. However, we failed to demonstrate correlations between changes in KL-6 levels during the first 6 months and subsequent ILD progression using different criteria. This observation in patients with SSc-ILD contrasted with the utility of serial KL-6 level measurements in patients with ILD associated with myositis or anti-synthetase syndrome, in which serial changes in KL-6 levels were useful to predict treatment response, long-term outcomes and relapse [31,32,33,34,35]. Our findings do not justify the serial measurement of serum KL-6 levels in patients with SSc-ILD.

A strength of this study included the measurement of KL-6 levels every 2 months for 2 years, since previous studies evaluating serial changes in KL-6 levels utilized samples obtained at baseline and only one follow-up timepoint. Kumánovics and colleagues measured serum KL-6 levels at baseline and 9 ± 22 months later in 158 patients with SSc and found that KL-6 levels decreased in 31 patients with SSc-ILD who were treated with CYC independent of the improvement or deterioration of their PFT parameters [17]. In another study using baseline and 12-month plasma samples from patients enrolled in a Scleroderma Lung Study II, KL-6 levels decreased in response to treatment with CYC and MMF for one year, while the patients assigned to receive MMF experienced the greatest decline in KL-6 levels [16]. This is the first study to investigate detailed fluctuations in serum KL-6 levels in SSc patients, but we failed to show the clinical relevance of serial measurements of serum KL-6 levels. Nevertheless, we believe that it is still worth evaluating the roles of KL-6 fluctuations in the pathogenic process of SSc-ILD.

One of the potential reasons for the lack of a correlation between short-term changes in KL-6 levels and subsequent ILD progression in our patients was a low frequency of patients with ILD progression: only 3% of the patients with SSc-ILD experienced a relative decline in FVC ≥ 10% over one year. On the other hand, in the Royal Brompton Hospital cohort involving 162 patients with SSc-ILD, 21% experienced a relative decline of ≥10% over one year [27]. A relative decline in FVC (mL) from baseline to 52 weeks was observed in 52 (18%) of the 288 patients with SSc-ILD assigned to the placebo group in the nintedanib trial (SENSCIS) [4], while a relative decline in FVC (%) from baseline to 48 weeks was observed in 14 (25%) of the 56 patients with SSc-ILD assigned to the placebo group in the tocilizumab trial (focuSSced) [5]. The patient characteristics were different among the studies, but the disease duration at entry in our study (mean 2.4 years) was comparable to the Royal Brompton Hospital cohort (median 41 months), the SENSCIS trial (median 3.5 years) and the focuSSced trial (mean 22.6 months). In terms of treatment, 46% of the Royal Brompton Hospital cohort received corticosteroids (prednisolone at a dosage of ≥1 mg/day) and/or immunosuppressive agents (CYC, mycophenolate, azathioprine or cyclosporine). The patients enrolled in the SENSCIS trial received stable treatment (≥6 months) with mycophenolate (49%) or methotrexate (5%) [4], while the use of immunomodulatory drugs was not permitted in the focuSSced trial [5]. In our study, 47% of the patients received immunomodulatory treatment, and eight patients (12%) were treated with tocilizumab, which was not used in the other patient populations. In this regard, in the focuSSced trial, a decline in FVC over 48 weeks was almost completely prevented in the tocilizumab group compared with the placebo group (−0.1% versus −6.4%) [5]. Since patients with multiple risk factors for ILD progression are likely to be treated in clinical practice, the low proportion of ILD progression in this study might have been influenced by the use of immunomodulatory drugs, which were shown to prevent a decline in pulmonary function [5,22,23,24]. In fact, our study failed to replicate the association of baseline KL-6 levels with the subsequent progression of SSc-ILD, which was shown in previous studies [8,15,16]. Since many patients with SSc-ILD with risk for progression are treated with immunomodulatory treatment and/or antifibrotic nintedanib recently in clinical practice, it might be difficult to use real-world data to evaluate the risk factors for ILD progression.

Another potential reason for the lack of a correlation included a decrease in serum KL-6 levels by treatment with CYC or MMF in patients with SSc-ILD, independent of the improvement or deterioration of pulmonary function [16,17]. However, no correlation between short-term changes in KL-6 levels and subsequent ILD progression was observed by the use of any immunomodulatory treatment as a confounding factor in the multivariable analysis.

We fully acknowledge that a substantial weakness of this study is the retrospective, two-center study design. The patient selection bias was inevitable because only the patients with a chest X-ray and a PFT available at SSc diagnosis, a KL-6 measurement every 2 months after SSc diagnosis and a follow-up period of ≥2 years were included. The use of a placebo group of clinical trials, rather than prospective cohorts, is useful to evaluate the utility of short-term trends in KL-6 levels in predicting the subsequent progression of ILD in patients with SSc-ILD, although it totally depends on the availability of serum samples obtained repeatedly at short intervals.

## 5. Conclusions

Serum KL-6 levels fluctuate in patients with SSc-ILD, but the current findings do not support the serial measurement of serum KL-6 levels in patients with SSc-ILD in clinical practice.

## Figures and Tables

**Table 1:** Baseline characteristics and immunomodulatory treatment during the first 2 years after diagnosis in SSc patients used for analyses.

Clinical Parameters	All SSc Patients (*n* = 110)	SSc-ILD Patients (*n* = 64) ^1^
Sex (female)	96 (87%)	57 (89%)
Age at study entry	54 ± 13	53 ± 13
Disease duration (years)	2.6 ± 2.0	2.4 ± 1.9
dcSSc	46 (42%)	33 (52%)
SSc-related autoantibodies		
Anti-topoisomerase I	45 (41%)	33 (52%)
Anticentromere	31 (28%)	10 (16%)
Anti-U1 RNP	11 (10%)	8 (13%)
Anti-RNAP III	3 (5%)	2 (3%)
Anti-U3 RNP	2 (2%)	0
Anti-Th/To	1 (1%)	1 (2%)
Current or past smoker	31 (28%)	21 (33%)
ILD	71 (65%)	64 (100%)
ILD extent shown on HRCT (%)	NA	20.7 ± 20.9
Extensive disease	NA	23 (36%)
FVC (% predicted) ^2^	97.5 ± 17.6	89.8 ± 15.3
DL_CO_ (% predicted) ^2^	78.5 ± 25.1	69.1 ± 21.8
CRP levels (mg/dL)	0.14 ± 0.28	0.16 ± 0.29
KL-6 levels (U/mL) ^2^	775 ± 857	994 ± 899
Any immunomodulatory treatment during the first 2 years after diagnosis	39 (35%)	30 (47%)

^1^ This cohort was selected from all SSc cohort based on the presence of ILD by HRCT and availability of follow-up PFT data at 2 years for the analysis of potential correlations of short-term KL-6 level changes with the subsequent progression of ILD. ^2^ *p* < 0.01 between all patients with SSc and patients with SSc-ILD. NA: not applicable; ILD: interstitial lung disease; dcSSc: diffuse cutaneous SSc; RNAP III: RNA polymerase III; RNP: ribonucleoprotein; HRCT: high-resolution computed tomography; FVC: forced vital capacity; DL_CO_: diffusing capacity for carbon monoxide; CRP: C-reactive protein, KL-6: Krebs von den Lungen-6.

**Table 2:** Simple regression analysis to identify the factors associated with variability of KL-6 levels over 2 years in 110 patients with SSc.

Clinical Parameters	β	*p*
Sex (female)	0.016	0.866
Age at study entry	0.033	0.733
Disease duration (years)	−0.186	0.052
dcSSc	0.198	0.038
Anti-topoisomerase I	0.241	0.011
Anticentromere	−0.256	0.007
Anti-U1 RNP	0.089	0.356
Anti-RNAP III	0.035	0.720
Current or past smoking	−0.065	0.611
ILD	0.462	<0.001
Disease extent shown on HRCT (%) ^1^	0.293	0.01
Extensive disease ^1^	0.358	0.001
FVC (% predicted)	−0.252	0.008
DL_CO_ (% predicted)	−0.413	<0.001
CRP levels (mg/dL)	0.104	0.279
KL-6 levels (U/mL)	0.369	<0.001
Any immunomodulatory treatment during the first 2 years after diagnosis	0.292	0.002

^1^*n* = 76. ILD: interstitial lung disease, dcSSc: diffuse cutaneous SSc, RNAP III: RNA polymerase III, RNP: ribonucleoprotein, HRCT: high-resolution computed tomography, FVC: forced vital capacity, DL_CO_: diffusing capacity for carbon monoxide, CRP: C-reactive protein.

**Table 3:** Baseline characteristics and immunomodulatory treatment between patients who experienced progression of ILD as defined by the OMERACT criteria ^1^ and those who did not.

Clinical Parameters	ILD Progression (*n* = 12)	No ILD Progression (*n* = 52)	*p*
Sex (female)	11 (92%)	46 (88%)	1.00
Age at study entry	52 ± 14	53 ± 13	0.77
Disease duration (years)	1.7 ±1.4	2.6 ± 2.0	0.17
dcSSc	8 (67%)	25 (48%)	0.34
SSc-related autoantibodies			
Anti-topoisomerase I	8 (67%)	25 (48%)	0.34
Anticentromere	2 (17%)	8 (15%)	1.00
Anti-U1RNP	2 (17%)	6 (12%)	0.64
Current or past smoker	4 (28%)	17 (36%)	1.00
ILD extent shown on HRCT (%)	29.6 ± 21.2	18.7 ± 20.4	0.041
Extensive disease	8 (67%)	15 (20%)	0.020
FVC (% predicted)	91.1 ± 19.6	89.5 ± 14.4	0.58
DL_CO_ (% predicted)	67.9 ± 27.0	69.4 ± 20.7	0.99
CRP levels (mg/dL)	0.24 ± 0.33	0.14 ± 0.29	0.14
KL-6 levels (U/mL)	1124 ± 682	964 ± 945	0.18
Any immunomodulatory treatment	5 (42%)	25 (48%)	0.76

^1^ A ≥ 10% relative decline in FVC or a ≥ 5% to <10% relative decline in FVC and a ≥ 15% relative decline in DL_CO_ over a 2-year period. ILD: interstitial lung disease, dcSSc: diffuse cutaneous SSc, HRCT: high-resolution computed tomography, FVC: forced vital capacity, DL_CO_: diffusing capacity for carbon monoxide, CRP: C-reactive protein.

**Table 4:** Correlations between baseline KL-6 levels or indices for short-term changes in KL-6 levels and ILD progression in 64 patients with SSc-ILD.

	Progression of ILD	
	OMERACT Criteria ^1^	PF-ILD ^2^
KL-6 levels at baseline	0.58	0.32
Regression coefficient over 6 months	0.83	0.78
A ratio of the KL-6 level at 6 months to the baseline	0.39	0.69
AUC above the standard line	0.52	0.59
AUC above the line of baseline KL-6 levels	0.77	0.31
Consecutive rise over 6 months	1.00	0.94

*p* values of individual univariate logistic regression analyses are shown. ^1^ A ≥ 10% relative decline in FVC or a ≥ 5% to <10% relative decline in FVC and a ≥ 15% relative decline in DL_CO_ over a 2-year period. ^2^ A ≥ 10% relative decline in FVC or a ≥ 5 to <10% relative decline in FVC in combination with any of the following: a ≥ 15% relative decline in DL_CO_, worsening respiratory symptoms or worsening radiological appearance. ILD: interstitial lung disease, OMERACT: Outcome Measures in Rheumatology, PF-ILD: progressive fibrosing ILD, AUC: area under the curve.

## Data Availability

Raw data are available from the corresponding author upon reasonable request.

## Supplementary Materials

The following are available online at https://www.mdpi.com/article/10.3390/diagnostics11112007/s1: Figure S1: A patient flow for a selection of SSc patients, Table S1: Multiple regression analysis to identify the independent factors associated with the variability of KL-6 levels over 2 years in 110 patients with SSc, Figure S2: FVC at baseline and after 2 years of follow-up in 64 patients with SSc-ILD, Table S2: Baseline characteristics and immunomodulatory treatment between patients who experienced progression of ILD defined by PF-ILD and those who did not, TableS3: Correlations between baseline KL-6 levels or indices for the short-term changes in KL-6 levels with ILD progression adjusted for immunomodulatory treatment in 64 patients with SSc-ILD.
