# Supplementary material for: Clinical Relevance of the Serial Measurement of Krebs von den Lungen-6 Levels in Patients with Systemic Sclerosis-Associated Interstitial Lung Disease
Source: Diagnostics (Basel). 2021 Oct 28;11(11):2007. doi: 10.3390/diagnostics11112007 (PMC8619247; doi:10.3390/diagnostics11112007)
Supplement: Supplementary file 1 [file diagnostics-11-02007-s001.zip › diagnostics-1393534-supplementary.pdf]

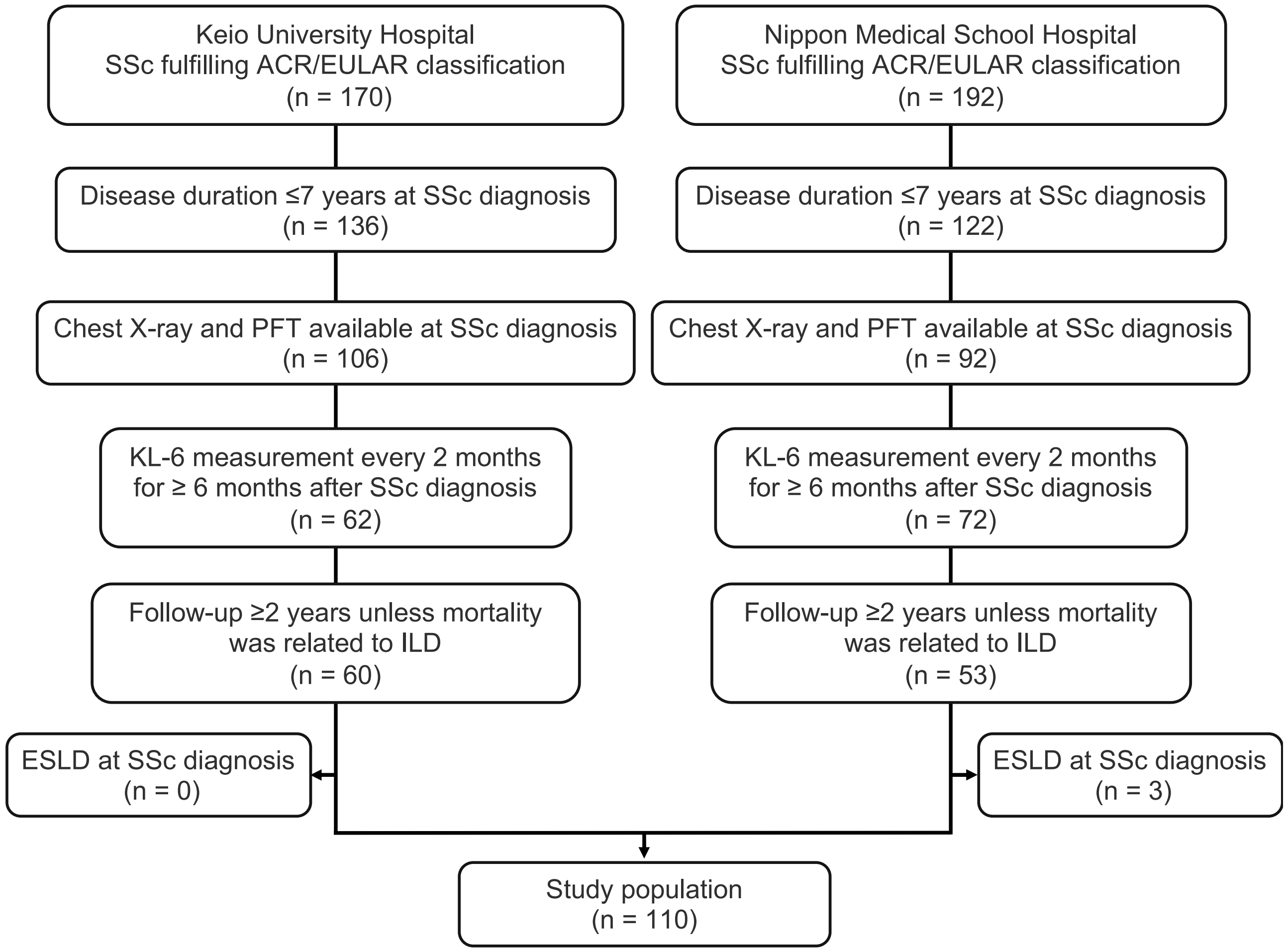

Supplementary Figure S1. A patient flow for selection of SSc patients.

ACR: American College of Rheumatology, EULAR: European League against Rheumatism, PFT: pulmonary function test, ESLD: end-stage lung disease.

Supplementary Table S1. Multiple regression analysis to identify the independent factors associated with variability in KL-6 levels over 2 years in 110 patients with SSc.

**Clinical parameters**	**Model #1**		**Model #2**		**Model #3**		**Model #4**		**Model #5**		**Model #6**	
	β	***P***	β	***P***	β	***P***	β	***P***	β	***P***	β	***P***
Female	0.098	0.43	0.039	0.78	0.095	0.45	0.11	0.37	0.052	0.71	0.11	0.38
Age	0.037	0.77	0.047	0.72	0.054	0.68	0.067	0.64	0.083	0.55	0.075	0.59
Smoking	0.028	0.83	0.024	0.85	0.042	0.74	0.012	0.93	0.006	0.96	0.025	0.84
dcSSc	0.061	0.69	0.026	0.86	0.016	0.92	0.11	0.43	0.078	0.56	0.068	0.62
Baseline KL-6 levels	0.061	0.68	0.025	0.87	0.051	0.73	0.059	0.69	0.020	0.89	0.048	0.74
Any immunomodulatory treatment	0.10	0.43	0.072	0.58	0.11	0.41	0.10	0.41	0.074	0.56	0.11	0.37
ILD extent shown on HRCT	0.28	0.063	0.18	0.23	0.22	0.14	0.27	0.073	0.16	0.29	0.21	0.15
Extensive disease												
Anti-topo I	0.14	0.37	0.16	0.28	0.15	0.32						
Anticentromere							-0.12	0.37	-0.15	0.29	-0.11	0.43
FVC	0.11	0.38					0.12	0.33				
DL_{CO}			-0.17	0.26					-0.18	0.23		
ILD					0.12	0.33					0.11	0.38

ILD; interstitial lung disease, dcSSc; diffuse cutaneous SSc, HRCT; high-resolution computed tomography, topo I; topoisomerase I, FVC; forced vital capacity, DL_{CO}; diffusing capacity for carbon monoxide.

Supplementary Table S1. Multiple regression analysis to identify the independent factors associated with variability in KL-6 levels over 2 years in 110 patients with SSc (continued).

Clinical parameters	Model #7		Model #8		Model #9		Model #10		Model #11		Model #12	
	β	***P***	β	***P***	β	***P***	β	***P***	β	***P***	β	***P***
Female	0.058	0.63	0.018	0.89	0.066	0.59	0.078	0.52	0.039	0.77	0.088	0.47
Age	0.074	0.55	0.075	0.55	0.082	0.51	0.11	0.40	0.11	0.40	0.11	0.43
Smoking	0.037	0.76	0.038	0.76	0.060	0.62	0.021	0.86	0.021	0.87	0.042	0.73
dcSSc	0.044	0.76	0.010	0.94	-0.006	0.97	0.10	0.45	0.070	0.59	0.057	0.67
Baseline KL-6 levels	0.017	0.90	-0.023	0.87	-0.005	0.97	0.008	0.95	-0.033	0.82	-0.008	0.96
Any immunomodulatory treatment	0.098	0.43	0.070	0.57	0.10	0.42	0.098	0.42	0.073	0.56	0.11	0.38
ILD extent shown on HRCT												
Extensive disease	0.38	0.008	0.29	0.040	0.32	0.019	0.38	0.008	0.28	0.048	0.31	0.022
Anti-topo I	0.17	0.24	0.19	0.19	0.18	0.22						
Anticentromere							-0.17	0.20	-0.17	0.20	-0.14	0.32
FVC	0.13	0.28					0.15	0.21				
DL_{CO}			-0.15	0.30					-0.15	0.28		
ILD					0.13	0.27					0.11	0.37

ILD; interstitial lung disease, dcSSc; diffuse cutaneous SSc, HRCT; high-resolution computed tomography, topo I; topoisomerase I, FVC; forced vital capacity, DL_{CO}; diffusing capacity for carbon monoxide

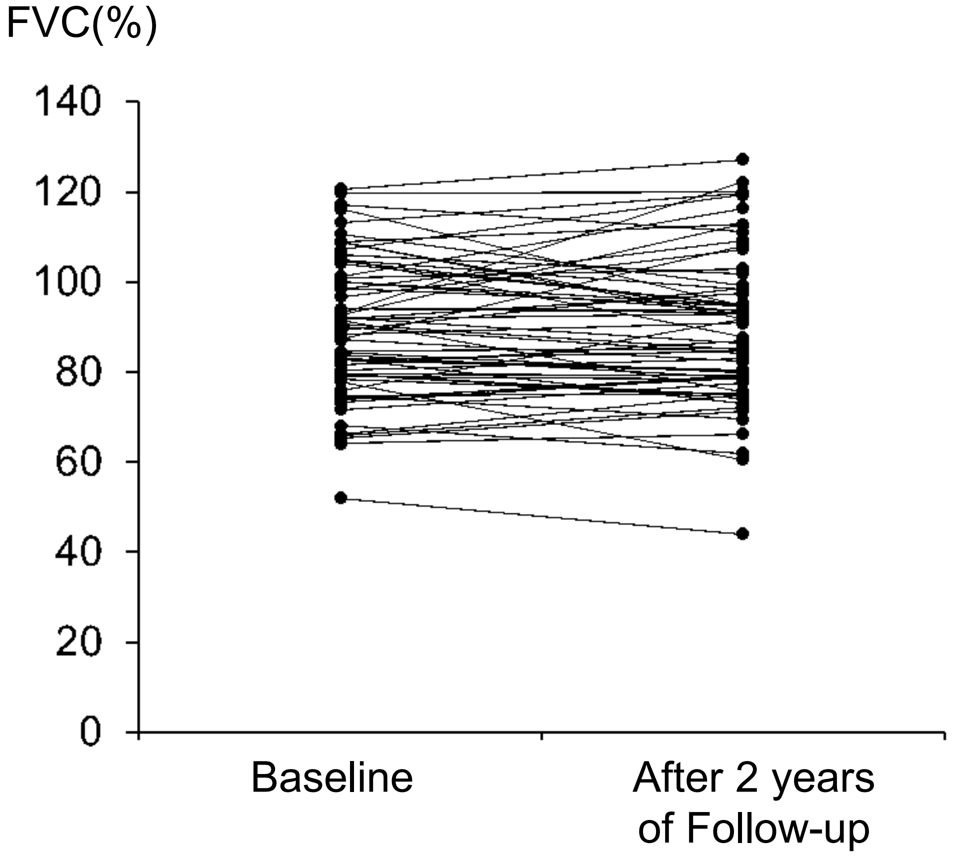

Supplementary Figure S2. FVC at baseline and after 2 years of follow-up in 64 patients with SSc-ILD.

Supplementary Table S2. Baseline characteristics and immunomodulatory treatment between patients who experienced progression of ILD defined by PF-ILD[1] and those who did not.

**Clinical parameters**	**ILD progression (n =22)**	**No ILD progression (n = 42)**	***P***
Sex (female)	19 (86%)	38 (90%)	0.68
Age at study entry	51 ± 14	55 ± 13	0.28
Disease duration (years)	2.1 ± 1.9	2.6 ± 2.0	0.38
dcSSc	14 (64%)	19 (45%)	0.20
SSc-related autoantibodies			
Anti-topoisomerase I	14 (64%)	19 (45%)	0.20
Anticentromere	2 (9%)	8 (19%)	0.47
Anti-U1RNP	2 (9%)	6 (14%)	0.70
Current or past smoker	9 (41%)	12 (29%)	0.40
ILD extent shown on HRCT (%)	25.5 ± 21.7	18.2 ± 20.2	0.15
Extensive disease	12 (55%)	11 (26%)	0.031
FVC (% predicted)	90.7 ± 17.8	89.4 ± 14.1	0.64
DL_{CO} (% predicted)	73.1 ± 29.5	67.0 ± 16.4	0.48
CRP levels (mg/dL)	0.15 ± 0.26	0.16 ± 0.32	0.66
KL-6 levels (U/mL)	1152 ± 930	911 ± 882	0.097
Any immunomodulatory treatment	11 (50%)	19 (45%)	0.80

[1] A ≥10% relative decline in FVC, or a ≥5 to <10% relative decline in FVC in combination with any of the following: a ≥15% relative decline in DL_{CO}, worsening respiratory symptoms, or worsening radiological appearance.

ILD; interstitial lung disease, dcSSc; diffuse cutaneous SSc, HRCT; high-resolution computed tomography, FVC; forced vital capacity, DL_{CO}; diffusing capacity for carbon monoxide, CRP; C-reactive protein

Supplementary Table S3. Correlations between baseline KL-6 levels or indices for short-term changes in KL-6 levels and ILD progression adjusted for immunomodulatory treatment in 64 patients with SSc-ILD.

	**Progression of ILD**	
	**OMERACT criteria[1]**	**PF-ILD[2]**
KL-6 levels at baseline	0.52	0.34
Regression coefficient over 6 months	0.78	0.74
A ratio of the KL-6 level at 6 months to the baseline	0.39	0.70
AUC above the standard line	0.46	0.63
AUC above the line of the baseline KL-6 level	0.77	0.32
Consecutive rise over 6 months	1.00	0.95

P values of individual univariate logistic regression analyses are shown.

[1] A ≥10% relative decline in FVC or a ≥5% to <10% relative decline in FVC and a ≥ 15% relative decline in DL_{CO} over a 2-year period.

[2] A ≥10% relative decline in FVC or a ≥5 to <10% relative decline in FVC in combination with any of the following: a ≥15% relative decline in DL_{CO}, worsening respiratory symptoms, or worsening radiological appearance.

ILD; interstitial lung disease, PF-ILD; progressive fibrosing ILD, AUC; area under the curve.
